# Depression and suicidality with VMAT2 inhibitors in tardive dyskinesia A signal detection from the FDA Adverse Events Reporting System

**DOI:** 10.1002/pcn5.79

**Published:** 2023-02-27

**Authors:** Yuma Yokoi, Hiroko Kashiwagi, Daisuke Funada, Shingo Yamashita, Chika Kubota

**Affiliations:** ^1^ Department of Educational Promotion, Clinical Research & Education Promotion Division, National Center Hospital National Center of Neurology and Psychiatry Tokyo Japan; ^2^ Department of Psychiatry, National Center Hospital National Center of Neurology and Psychiatry Tokyo Japan; ^3^ Department of Forensic Psychiatry, National Center Hospital National Center of Neurology and Psychiatry Tokyo Japan

Tardive dyskinesia (TD) is a neurological movement disorder observed after long‐term exposure to dopamine‐blocking agents such as antipsychotics. TD has become common in mood disorders, along with recent expanding indications of antipsychotics. Unfortunately, reduction or cessation of antipsychotics sometimes does not ameliorate TD symptoms with the risk of relapse.^1^

Deutetrabenazine and valbenazine are the two vesicular monoamine transporter 2 (VMAT2) inhibitors approved in the United States in 2017. Given that the basal ganglia and dopaminergic pathways play a significant role in hyperkinetic movement disorders, VMAT2 seems effective. However, VMAT2 inhibition is known to cause severe serotonin^2^ and dopamine depletion. Occurrence of suicide‐related events in the phase 3 study of tetrabenazine, another VMAT2 inhibitor approved for Huntington's chorea, resulted in the black‐box warning^3^ in the US package insert. Although deutetrabenazine and valbenazine did not show apparent depression or suicide during clinical trials, we explored possible signals of suicide and depression in VMAT2 inhibitors from postmarketing safety data.

We searched the spontaneous adverse events reports by the US Food and Drug Administration (FDA), known as the FDA Adverse Events Reporting System (FAERS). Data comes from Q1 2004 to Q3 2021 with OpenVigil,^4^ a web‐based tool to acquire and clean the FAERS database. Its latest version is 2.1‐MedDRA‐v24, using the Medical Dictionary for Regulatory Activities (MedDRA) version 24.0 to label adverse events (AEs). Also, we found a similar database called VigiBase but abandoned it because all TD reports are from the United States. We used the Standardized MedDRA Queries (SMQ) “depression and suicide/self‐injury” to detect the related preferred terms (PTs). Plus, we checked the high‐level term (HLT) “death and sudden death” for potential suicide cases.

We calculated the proportional reporting ratio (PRR) and the reported odds ratio (ROR) for the disproportionality analysis. Evans developed the PRR and the criteria for a signal: three or more cases, PRR at least two, and *χ* ^2^ at least four.^5^ ROR measures the magnitude of association between exposure to a pharmaceutical and the odds of a specific outcome occurring^6^ and is significant when the lower limit of the 95% confidence interval is >1.0. Although OpenVigil shows the PRR and ROR, we performed all analyses using STATA version 14.^7^

Results are shown in Table 1. Demographics show more female cases than males, and the mean age was similar among drugs. PT drug ineffective was the most frequently reported AE.

**Table 1:** Analyses of FAERS data of tetrabenazine, deutetrabenazine, and valbenazine.

Demographics		Tetrabenazine		Deutetrabenazine		Valbenazine	
Number of reports		4192		1677		10,230	
Female (%)		2388 (57.0)		1053 (62.8)		5134 (50.2)	
SAE (%)		2529 (60.3)		512 (30.5)		2086 (20.4)	
Mean age ± SD (cases)		55.2 ± 20.6 (2085)		58.3 ± 16.3 (677)		59.9 ± 13.7 (4419)	
Major AEs reported		Events	Counts (%)	Events	Counts (%)	Events	Counts (%)
		Drug ineffective	1242 (29.6)	Depression	596 (35.5)	Drug ineffective	1107 (10.8)
		Death	1190 (28.4)	Drug ineffective	394 (23.5)	Somnolence	971 (9.5)
		Depression	1080 (25.8)	Fatigue	383 (22.8)	Fatigue	728 (7.1)
Major SAEs reported		Events	Counts	Events	Counts	Events	Counts
		Death	818 (19.5)	Death	71 (4.2)	Death	322 (3.1)
		Depression	155 (3.7)	Depression	62 (3.7)	Hospitalization	246 (2.4)
		Hospitalization	142 (3.4)	Suicide ideation	62 (3.7)	Fall	185 (1.8)

Disproportionality analysis			Tetrabenazine		Deutetrabenazine		Valbenazine
SMQ “Depression and suicide/self‐injury”	*χ* ^2^		520.874		236.891		20.584
	PRR		2.308 [2.152–2.476]		2.397 [2.150–2.672]		0.833 [0.770–0.902]
	ROR		2.552 [2.348–2.773]		2.669 [2.344–3.038]		0.823 [0.757–0.895]
HLT “death and sudden death”	*χ* ^2^		1840.687		2.464		66.125
	PRR		3.862 [3.633–4.106]		0.829 [0.660–1.041]		0.653 [0.588–0.724]
	ROR		4.565 [4.230–4.926]		0.821 [0.648–1.042]		0.640 [0.575–0.713]

Abbreviations: AE, adverse event; FAERS, US Food and Drug Administration (FDA) Adverse Events Reporting System; HLT, high level terms; PRR, proportional reporting ratio; ROR, reported odds ratio; SAE, serious adverse event; SD, standard deviation; SMQ, standard MedDRA queries.

Disproportionality analysis of SMQ showed PRR and ROR significantly more than 2 in etrabenazine and deutetrabenazine and <1 in valbenazine. Also, regarding HLT, PRR and ROR were more significant than 2 in tetrabenazine and <1 in deutetrabenazine and valbenazine.

Evans' criteria^5^ indicate the SMQ signal in tetrabenazine and deutetrabenazine, while the HLT signal was only seen in tetrabenazine. The difference is presumably due to the proportion of serious reports or target populations. A recent cross‐sectional study showed that tetrabenazine was not associated with depression (odds ratio [OR] = 0.71, *P* = 0.016) and suicidal ideation (OR = 0.57, *P* = 0.07) in patients with former depression, while a trend of higher incidence of depression (OR = 1.59, *P* = 0.18) or suicidality (OR = 1.43, *P* = 0.66) was seen in patients without former depression.^8^

The SMQ signal in deutetrabenazine was the same as that in tetrabenazine. Deutetrabenazine is structurally identical to tetrabenazine except that several hydrogen atoms are replaced by deuterium, which enables slower degradation, longer half‐life, and infrequent dosing.^9^ Interestingly, the boxed warning of depression in deutetrabenazine is merely for Huntington's disease. Among reports with valbenazine, antidepressants were more prevalent in patients with SMQ, but it is indistinguishable whether antidepressants were used for AEs or background illnesses. Pharmacologically, metabolites of tetrabenazine and deutetrabenazine may have broader effects on dopaminergic and serotoninergic receptors than valbenazine, whose activity is mainly limited to VMAT2.^8^ In the long run, VMAT2 selectivity might ameliorate the motor and psychopathological symptoms related to monoamine deficiency.^10^

This study has limitations, as we have already discussed. Results from spontaneous reports cannot be conclusive because of unavoidable reporting bias and influence by prior information such as black box warnings and results of clinical trials. It is unclear whether the demographics represent the population, thus our analyses did not adjust for covariates. Also, the PRRs and RORs obtained differ across medications because they depends on how often and when each drug reports AEs. In addition, FAERS does not show the details of individual cases, so we can only look at the numbers and see the trends.

Despite the limitations of this study, it will be of value in detecting signals and continuing pharmacovigilance to guide further research and patient safety.

## AUTHOR CONTRIBUTION

Yuma Yokoi conceived the concept, developed the theoretical formalism, and performed the analytic calculations. Hiroko Kashiwagi, Daisuke Funada, Shingo Yamashita, and Chika Kubota contributed to the final version of the manuscript. Chika Kubota supervised the project.

## CONFLICT OF INTEREST STATEMENT

The authors declare no conflict of interest.

## ETHICS APPROVAL STATEMENT

Ethical approval was waived due to de‐identified public data. This research follows the Declaration of Helsinki.

## PATIENT CONSENT STATEMENT

Ethical approval was waived due to de‐identified public data.

## CLINICAL TRIAL REGISTRATION

N/A.

## Data Availability

N/A.
